# Pancreatic Satellite Cells Derived Galectin-1 Increase the Progression and Less Survival of Pancreatic Ductal Adenocarcinoma

**DOI:** 10.1371/journal.pone.0090476

**Published:** 2014-03-04

**Authors:** Dong Tang, Jingqiu Zhang, Zhongxu Yuan, Jun Gao, Sen Wang, Nianyuan Ye, Ping Li, Sujun Gao, Yi Miao, Daorong Wang, Kuirong Jiang

**Affiliations:** 1 Department of General Surgery, Subei People's Hospital of Jiangsu Province (Clinic Medical College of Yang Zhou University), Yangzhou, Jiangsu Province, China; 2 Department of Digestive System, Subei People's Hospital of Jiangsu Province (Clinic Medical College of Yang Zhou University), Yangzhou, Jiangsu Province, China; 3 Department of General Surgery, Anhui no. 2 Provincial People's Hospital, Hefei, Anhui Province, China; 4 College of Clinical Medicine, Nanjing Medical University (the First Affiliated Hospital of Nanjing Medical University), Nanjing, Jiangsu Province, China; 5 Department of General Surgery, the First Affiliated Hospital of Nanjing Medical University, Nanjing, Jiangsu Province, China; Rutgers - New Jersey Medical School, United States of America

## Abstract

**Background:**

Galectin-1, a member of carbohydrate-binding proteins with a polyvalent function on tumor progression, was found strongly expressed in pancreatic satellite cells (PSCs), which partner in crime with cancer cells and promote the development of pancreatic ductal adenocarcinoma (PDAC). We evaluated the effects of PSCs derived Galectin-1 on the progression of PDAC, as well as the tumor establishment and development in mouse xenografts.

**Methods:**

The relationship between immunohistochemistry staining intensity of Galectin-1 and clinicopathologic variables were assessed in 66 PDAC tissues, 18 chronic pancreatitis tissues and 10 normal controls. The roles of PSCs isolated from PDAC and normal pancreas on the proliferative activity, MMP2 and MMP9 expression, and the invasion of CFPAC-1 in the co-cultured system, as well as on the tumor establishment and development in mouse xenografts by mixed implanting with CFPAC-1 subcutaneously were evaluated.

**Results:**

Galectin-1 expression was gradually increased from normal pancreas (negative), chronic pancreatitis (weak) to PDAC (strong), in which Galectin-1 expression was also increased from well, moderately to poorly differentiated PDAC. Galectin-1 staining intensity of pancreatic cancer tissue was associated with increase in tumor size, lymph node metastasis, perineural invasion and differentiation and UICC stage, and served as the independent prognostic indicator of poor survival of pancreatic cancer. In vitro and in vivo experiments indicated that TGF-β1 upregulated Galectin-1 expression in PSCs, which could further promotes the proliferative activity, MMP2 and MMP9 expression, and invasion of pancreatic cancer cells, as well as the tumor establishment and growth.

**Conclusion:**

Galectin-1 expression in stromal cells of pancreatic cancer suggests that this protein plays a role in the promotion of cancer cells invasion and metastasis and provides a therapeutic target for the treatment of pancreatic cancer.

## Introduction

Pancreatic ductal adenocarcinoma (PDAC) is one of the most lethal human malignancies with occult onset and poor prognosis[1]. The rapid clinical deterioration commonly observed in patients with pancreatic cancer has been ascribed to the aggressive bionomics of PDAC which spread to other organs quickly with little time to intervene, and ineffectiveness of systemic therapies available for patients with advanced disease[2], [3]. However, even patients with pancreatic cancer who undergo surgery with clean microscopic surgical margins (R0 resection) and adjuvant chemotherapy, the median survival rate is approximately 2 years, with a 5-year survival of less than 20% during last 40 years' research[3], [4], [5]. Sufficient knowledge of the cellular function coupled to the distinctive anatomical features of pancreatic cancer would give hope for developing new methods of early detection, screening and diagnostic, as well as the treatment opportunities. Hithreto, increasing evidences had showed that pancreatic satellite cells (PSCs), a main source of the stromal of pancreatic cancer, partnered in crime with cancer cells and promoted the development of pancreatic cancer [6], [7]. While the mechanism and the responsiveness media of interaction between PSCs and pancreatic cancer cells still remains to reveal, which will help the formulation of proper treatment plans.

Galectin-1, a member of carbohydrate-binding proteins with an affinity for β-galactoside, was found strong expressed in the isolated activated PSCs[8], [9]. Galectin-1 is widely distributed in many normal and pathological tissues and appears to be functionally polyvalent, such as regulating cell proliferation, differentiation and apoptosis, mediating tumor transformation, growth and so on[10], [11], [12], [13]. It is widely reported that Galectin-1 is overexpressed in many different types of digestive system tumors, including gastric cancer, colorectal cancer, hepatocellular carcinoma and so on[10], [14], [15]. However, there are relatively few reports associated with this theme in pancreatic cancer. Galectin-1 was revealed with low abundance in the normal pancreas[16], [17], moderately to intensely expressed in fibroblasts of chronic pancreatitis samples[16], and strongly expressed in the stroma surrounding the cancer mass but negative in cancer cells[18]. Fibroblasts in the area of fibrosis in chronic pancreatitis and of desmoplastic reaction associated with pancreatic cancer are now recognized as activated PSCs[19], [20]. So the Galectin-1 in the activated PSCs of stromal may be has an important role on the development of pancreatic cancer.

The aim of this study was to detect the expression of PSCs derived Galectin-1 and its correlation with clinical and histopathological parameters of pancreatic cancer and chronic pancreatitis tissues, and then evaluated the effect of Galectin-1 expression of activated PSC on pancreatic tumor establishment and development, in subcutaneous xenografts mouse models.

## Materials and Methods

### Patients and Pancreatic Tissues

66 pancreatic cancer, 18 chronic pancreatitis and 10 normal pancreatic tissues were included in this study. The pancreatic cancer patients comprised 45 men and 21 women with a median age of 55 years (range, 37–83 years), and the chronic pancreatitis patients comprised 13 men and 5 women with a median age of 54.5 years (range, 27–71 years). The clinicopathologic characteristics of the patients are described in Table 1 and Table 2. 10 normal pancreatic tissue samples were obtained from partial pancreases resected for bile duct cancer, or duodenal ampulla cancer, as control tissues. Survival was measured from the time of pancreatic resection until death and follow-up data for all cases were available. The median overall survival time of patients with pancreatic cancer was 16.2 months (range, 4.7–78 months). No patients had received chemotherapy or radiotherapy before operations. The tissues adjacent to the specimens were evaluated histologically according to the criteria of the World Health Organization and tumor pathological stage was classified according to the International Union Against Cancer Classification (UICC).

**Table 1:** Galectin-1 intensity of staining and clinicopathologic characteristics of the 18 patients with chronic pancreatitis and 66 patients with pancreatic cancer.

Characteristics	Case n	Galectin-1 intensity			?2	*P* value
		Weak (+) n(%)	Moderate (++) n(%)	Strong (+++) n(%)		
**Chronic pancreatitis**						
Age, y						
≥55	10	1(10)	6(60)	3(30)	4.548	0.103
<55	8	3(37.5)	1(12.5)	4(50)		
Sex						
Male	13	3(23.1)	5(38.5)	5(38.5)	0.02	0.99
Female	5	1(20)	2(40)	2(40)		
Alcohol-drinking categories						
Low risk	4	3(75)	1(25)	0(0)	10.018	0.04
Moderate risk	6	1(16.7)	3 (50)	2(33.3)		
High risk	8	0(0)	3(37.5)	5(62.5)		
Smoking status						
Never	6	3(50)	2(33.3)	1(16.7)	5.920	0.205
Past	8	1(12.5)	4(50)	3(37.5)		
Current	4	0(0)	1(25)	3(75)		
**Pancreatic cancer**						
Age, y						
<55	29	10(34.5)	6(20.7)	13(44.8)	2.355	0.308
≥55	37	9(24.3)	14(37.8)	14(37.8)		
Sex						
Male	45	12(26.7)	14(31.1)	19(42.2)	0.311	0.856
Female	21	7(33.3)	6(28.6)	8(38.1)		
pT category						
pT1/pT2	19	10(52.6)	5(26.3)	4(21.1)	7.981	0.018
pT3/pT4	47	9(19.1)	15(31.9)	23(48.9)		
pN category						
pN0	27	12(44.4)	8(29.6)	7(25.9)	6.405	0.041
pN1	39	7(17.9)	12(30.8)	20(51.3)		
PNI						
Negative	34	13(38.2)	6(17.6)	15(44.1)	6.057	0.048
Positive	32	6(18.8)	14(43.8)	12(37.5)		
UICC stage						
I	17	9(52.9)	5(29.4)	3(17.6)	9.348	0.009
II	49	8(16.3)	19(38.8)	22(44.9)		
Differentiation						
well	10	6(60)	1 (10)	3(30)	11.532	0.021
moderately	24	8(33.3)	10(41.7)	6(25)		
Poorly	32	5(15.6)	9(28.1)	18(56.3)		
Alcohol-drinking categories						
Low risk	35	10(28.6)	12(34.3)	13(37.1)	4.443	0.349
Moderate risk	12	2(16.7)	2(16.7)	8(66.7)		
High risk	19	7(36.8)	6(31.6)	6(31.6)		
Smoking status						
Never	34	10(29.4)	12(35.3)	12(35.3)	4.94	0.293
Past	16	2(12.5)	5(31.3)	9(56.3)		
Current	16	7(43.8)	3(18.8)	6(37.5)		

UICC, International Union Against Cancer; PNI, perineural invasion.

**Table 2:** Univariate and multivariate analysis of conventional prognostic factors and stromal galectin-1 expression in the patients with pancreatic cancer.

Characteristics	Univariate		Multivariate	
	HR (95% CI)	*P*	HR (95% CI)	*P*
Age (≥55 vs. <55 years)	1.157(0.693–1.933)	0.576		
Sex (female vs. male)	0.636(0.376–1.074)	0.09		
pT category (pT3/pT4 vs. pT1/pT2)	2.538(1.318–4.887)	0.005	2.609(1.042–6.53)	0.041
pN category (pN1 vs. pN0)	2.754(1.641–4.623)	0.000	2.443(1.174–5.084)	0.017
PNI (positive vs. negative)	1.137(0.677–1.911)	0.627		
UICC stage (III+IV vs. I+II)	3.993(2.277–7.002)	0.000	4.202(0.893–19.774)	0.069
Differentiation				
Moderately vs. well	1.683(0.770–3.675)	0.192		
Poorly vs. well	5.106(2.256–11.554)	0.000	1.130(0.234–5.465)	0.879
Galectin-1 intensity				
Moderate vs. Weak	2.699(1.383–5.266)	0.004	1.773(0.796–3.949)	0.161
Strong vs. Weak	7.045(3.447–14.399)	0.000	4.346(1.94–9.733)	0.000

### Ethics statements

All patients have given informed consent for their participation in the study, which was approved by the Ethical Committee of Nanjing Medical University. Every participant provides their written informed consent to participate in this study, and the attachment is the formwork of Letter of Information and Consent for Participants. We reserved all the copy of written consent of participants in our laboratory and can be obtained all the time. The participants signed the Letter of Information and Consent, and each hold and saved one copy of the informed consent. Ethics committees approved this consent procedure, which has been record in the Consent Form of Ethical Committee.

All animal experiments were carried out according to the guidelines of the Experimental Animal Center Institutional Committee of Yangzhou University for Care and Use of Laboratory Animals. They were approved by the Institutional Animal Care and Research Advisory Committee of the Yangzhou University. Nude (CD1 nu/nu) male mice (Yangzhou University Comparative Medicine Center, License No: SCXK(Su) 20070009) were housed in cages (4 mice per cage) under conditions of constant photoperiod (12-hour light/12-shour dark) with free access to food and water. All the animals were scarified by nembutal overdose.

### Immunohistochemical staining and Evaluation

Immunohistochemical staining for Galectin-1 was performed as previously described[21]. Briefly, tissue sections were deparaffinized and rehydrated in PBS. Following antigen retrieval with the target retrieval solution, endogenous peroxidase activity was blocked by incubation with 0.3% hydrogen peroxide, and then sections were blocked in 10% normal goat serum. Sections were then incubated with a mouse monoclonal anti-Galectin-1 (1∶200; sc-166618, Santa Cruz Biotechnology, Inc., Santa Cruz, CA, USA) overnight at 4°C, followed by a biotinylated rabbit anti-mouse IgG antibody (1∶200; Vector Laboratories Inc., Burlingame, CA, USA) and then incubated with peroxidase-conjugated streptavidin (Boster, Wuhan, China.). For negative controls, primary antibody was replaced with nonspecific rabbit IgG. The results of immunohistochemical staining were interpreted by two experienced pathologists and the mean density of staining was calculated using the ImagePro Plus 6.0 software (ImagePro, Bethesda, MD, USA). Galectin-1 staining intensity was scored semiquantitatively as follows: no positive cells  = 0 (negative, -); 1–29% positive  = 1 (weak, +); 30–60% positive  = 2 (moderate, ++) and >60% positive  = 3 (strong, +++).

### Cells and Culture Conditions

Human PSCs were isolated from fresh normal pancreas and pancreatic cancer surgical specimens using the out-growth method[20], [22]. The PSCs cell type was confirmed by immunohistochemisty staining for α-SMA and by morphology (stellate-like or spindle-shaped cells)[8], [20], [23]. Passage numbers 2 to 5 of primary cultured PSCs were used for all of our experiments. Cells were maintained as previously described[24]. Pancreatic cancer cell lines (BxPC-3, SW1990,CFPAC-1 and PANC-1) was grown in DMEM with 10% FBS.

### Quantitative Reverse Transcription-Polymerase Chain Reaction (qRT-PCR)

Total RNA was extracted from the cultured cells using Trizol reagent (Invitrogen, Beijing, China) treatment according to the manufacturer's instructions. QRT-PCR was performed using a SYBR Premix Ex Taq Reverse Transcription-PCR kit (TaKaRa, Shiga, Japan). The following primers were used: Galectin-1 forward 5′- GAGGTGGCTCCTGACGCTAA -3′ and reverse 5′-CCTTGCTGTTGCACACGATG -3′, and β-actin forward 5′- AGAAAATCTGGCACCACACC-3′ and reverse 5′- TAGCACAGCCTGGATAGCAA-3′. β-actin was used as an internal control for comparison of the data. Quantitative PCR was performed using the ABI PRISM 7500 Fast Real-Time PCR System (Applied Biosystems, Carlsbad, CA, USA), and the comparative Ct method was used to assess relative changes in mRNA levels between two samples. All samples were run in triplicate.

### Western Blotting Analysis

Western blotting was performed as previously described[25]. Briefly, cells were lysed in SDS buffer and 100 µg total cellular protein was separated on 10% or 10–20% gradient SDS-polyacrylamide gels, transferred to PVDF, and incubated with mouse anti- Galectin-1, MMP2, MMP9 antibody (1∶500) overnight at 4°C. After incubation with peroxidase-conjugated rabbit anti-mouse IgG antibody (Cell Signaling Technologies, Beverly, MA, USA), proteins were visualized using ECL (GE Healthcare, Chalfont St. Giles, UK). α-Tubulin was used as a loading control.

### Flow cytometric analysis

S-phase fraction (cell cycle analysis) was carried out according to the method described by Tripathi[26]. CFPAC-1 cells were seeded in Transwell insert (0.4 µm pore size with polycarbonate membrane) and co-cultured with hCaPSC or hNPSC for 24 hours. After digestion and centrifugation, CFPAC-1 cells were washed twice and then fixed in 70% ethanol. Tubes containing the cell pellets were stored at 4°C for at least 24 hours. Cells were then centrifuged at 100×g for 10 minutes, and the supernatant was discarded. Pellets were washed twice stained with PI in the presence of RNase A for 45 minutes in dark. Samples were analyzed on a BD FACSCalibur flow cytometer (Beckman Coulter, Inc., Fullerton, CA) using 488 nm excitation wavelength. Ten thousand cells were analyzed per sample. Normal CFPAC-1 cells were used as the diploid reference standard.

### In Vitro Invasion Assay

Matrigel invasion assay was used to assess the ability of CFPAC-1 cells to penetrate the ECM in the presence of hCaPSC or hNPSC. First, the lower compartment was implanted with 2×10^5^ hCaPSCs or hNPSCs in complete medium for 12 hours. After PSCs adhered, the medium was changed to a serum-free medium. During the time, the upper chambers was coated with diluted Matrigel (1 mg/mL) (356243; BD Company, Bedford, Mass) and incubated at 37-C in 5%CO2 for 3 hours. After trypsinization, CFPAC-1 was suspended in serum-free medium in a concentration of 2×10^5^ cells/well and immediately placed onto the upper compartment. After 24 hours of incubation, the noninvading cells were removed from the upper surface of the membrane by wiping with cotton-tipped swabs. Cells on the lower surface of the membranewere stained with 0.1% crystal violet for 10 minutes and photographed. After that, crystal violet is bleached using 500 µL of 33% acetic acid. Absorbance was determined in a microtiter plate reader at 570 nm.

### In vivo Model of Animals Experiment

All animal experiments were carried out according to the guidelines of the Experimental Animal Center Institutional Committee of Yangzhou University for Care and Use of Laboratory Animals. They were approved by the Institutional Animal Care and Research Advisory Committee of the Yangzhou University. Nude (CD1 nu/nu) male mice (Yangzhou University Comparative Medicine Center, License No: SCXK(Su) 20070009) were housed in cages (4 mice per cage) under conditions of constant photoperiod (12-hour light/12-shour dark) with free access to food and water. Xenografts (n = 5/group) were established by implanting the human pancreatic cell line 1×10^6^ CFPAC-1 cells with or without 5×10^5^ PSCs (hCaPSC or hNPSC), subcutaneously (SC) on both flanks, using a 25-gauge needle. During the experiments, tumor size was determined with a caliper, according to the formula: length × width × depth×0.5236 according to the method described by Spector[27]. 30 days after the xenografts were established, all the animals were scarified by nembutal overdose in condition that no mortality was observed.

### Statistical Analysis

Values are expressed as means ± SE. One way ANOVA and *t*-test were used to look for differences between groups. The χ2 and Fisher's exact test were used to analyze the correlation between PSCs derived Galectin-1 expression and clinicopathologic characteristics seen in the immunohistochemical study. Univariate survival analysis was performed according to Kaplan-Meier; differences in survival curves were assessed with the log rank test. Multivariate analysis was performed using Cox's proportional hazard models. *P*-values are two-sided, *p*≤0.05 was considered statistically significant. All statistical analyses were performed using SPSS 13.0 software.

## Results

### Galectin-1 Staining and Association with Clinicopathologic Characteristics

**Figure 1:**
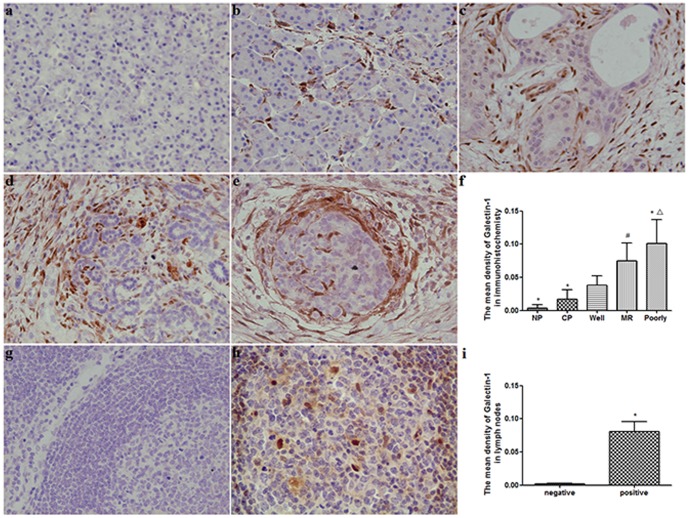
Characterization of Galectin-1 expressed in pancreatic disease. (*a*) Negative Galectin-1 expression in normal pancreas tissue. (*b*) Weak Galectin-1 expression in the stromal of chronic pancreatitis tissue. (*c–e*) Strong Galectin-1 expression in stromal cells of pancreatic cancer tissue, and increased from well (*c*), moderately (*d*) to poorly (*e*) differentiated pancreatic cancer. (*f*) Quantification of mean density of Galectin-1 expression in different pancreatic tissues. *p<0.01 *vs*. well, ^#^ *p*<0.05 *vs*. well, ^△^ *p*<0.05 *vs*. MR. NP, normal pancreas; CP, chronic pancreatitis; Well, Well differentiated pancreatic cancer; MR, moderately differentiated pancreatic cancer; Poorly, poorly differentiated pancreatic cancer. Original magnification: ×200. (*g*) Galectin-1 expression was negative in non-metastatic peripancreatic lymph nodes. (*h*) Galectin-1 positive staining was found in metastatic peripancreatic lymph nodes. (*i*) Quantification of mean density of Galectin-1 expression in non-metastatic and metastatic peripancreatic lymph nodes. *P<0.01 *vs*. negative.

To evaluate the relationship between Galectin-1 expression and clinicopathologic variables in chronic pancreatitis and PDAC, immunohistochemistry staining was performed in 66 PDAC tissues, 18 chronic pancreatitis tissues and 10 normal controls. The results indicated that the expression of Galectin-1 increased gradually from normal pancreas, chronic pancreatitis to PDAC (Table 1, Figure 1). Galectin-1 was negative in normal pancreas, whereas weak or moderate staining was mainly observed in the mesenchymal of chronic pancreatitis tissues (Figure 1 *a,b*). In chronic pancreatitis group, the Galectin-1 staining intensity in stromal cells was only significantly associated with alcohol-drinking (p = 0.04) (Table 1, Figure 1). In PDAC group, Galectin-1 expression was mainly detected in the stromal cells of all the pancreatic cancer (Figure 1 *c-e*). The Galectin-1 staining intensity was not obviously observed in cancer cells. The Galectin-1 staining intensity in stromal cells of PDAC were weak in 19 (28.8%), moderate in 20 (30.3%), strong in 27 (40.9%) cases. Galectin-1 staining intensity in stromal cells was significantly associated with increase in tumor size (pT3/pT4 vs. pT1/pT2, p = 0.018), perineural invasion (Positive vs. Negative p = 0.048), tumor stage (stage II vs. stage I, p = 0.007), differentiation (p = 0.021) and presence of lymph node metastasis (pN1 vs. pN0, p = 0.041), this association was not observed with other parameters (Table 1). A significant correlation was found between staining intensities of Galectin-1 in cancer-associated stromal cells and the differentiation of PDAC, the staining intensities of Galectin-1 in poorly-differentiated tissues was markedly stronger than that in well or moderately differentiated tissues (p = 0.0008, p = 0.0202, respectively), and also the staining intensities of Galectin-1 in moderately-differentiated tissues was higher than that in well-differentiated tissues (p = 0.0102) (Table 1, Figure 1 *c-f*). The correlation between Galectin-1 expression and the clinicopathological variables in patients with pancreatic cancer is summarized in Table 1. In addition, Galectin-1 was negative in non-metastatic peripancreatic lymph nodes and positive in tumor metastatic peripancreatic lymph nodes (Figure 1 *g-i*). The results implied that in chronic pancreatitis the Galectin-1 expression was associated with the alcohol-drinking, but in PDAC Galectin-1 expression was obviously related with tumor biological characteristics.

### Survival Analysis

**Figure 2:**
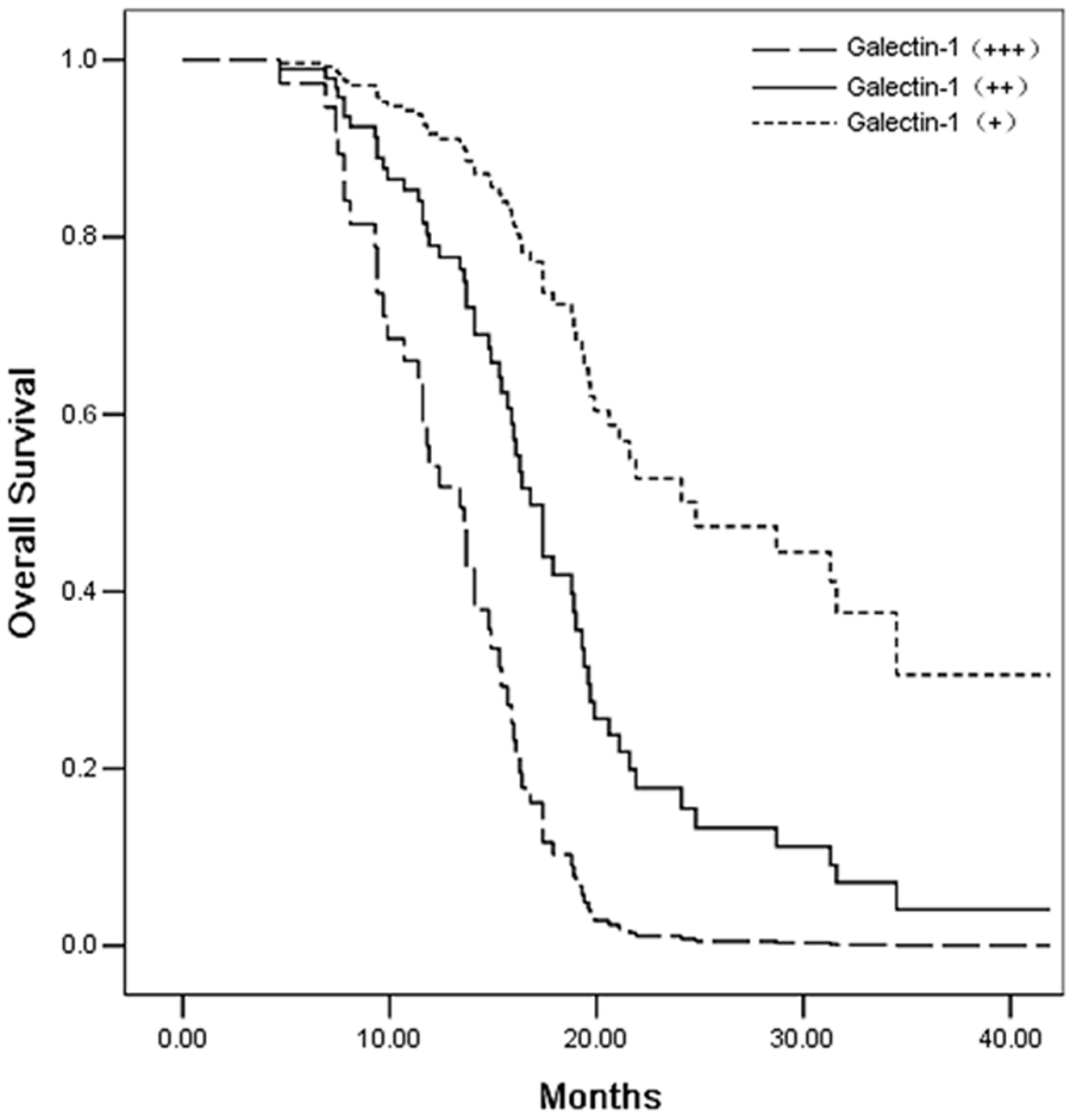
Kaplan-Meier survival curve for 66 pancreatic cancers with galectin-1 staining intensity in tumor associated stromal cells.

In order to evaluate Galectin-1 expression on the survival of patients with PDAC, univariate and multivariate analysis was performed. Univariate analysis using the log-rank test revealed adverse influences on survival with respect to tumor size (pT category, P = 0.005), lymph node involvement (pN category, p = 0.000), histology grade (Poorly differentiation, p = 0.000), tumor stage (p = 0.000), and Galectin-1 (p = 0.000) staining intensity in cancer-associated stromal cells. Cases with a strong or moderate Galectin-1 staining intensity in PDAC showed a significantly shortened mean survival time compared to the cases with weaker staining (Galectin-1+++ vs. Galectin-1+, p = 0.000; Galectin-1++ vs. Galectin-1+, p = 0.004) (Table 2, Figure 2). Furthermore, the multivariate survival analysis indicated that staining intensities of Galectin-1 in cancer-associated stromal cells (p<0.001), pT category (pT3/pT4) (p = 0.041) and pN category (pN1) (p = 0.017) remained significant independent prognostic factors (Table 2).

### Human PSCs Isolation and the Detection of Galectin-1 Expression in PSCs and Pancreatic Cancer Cell Lines

**Figure 3:**
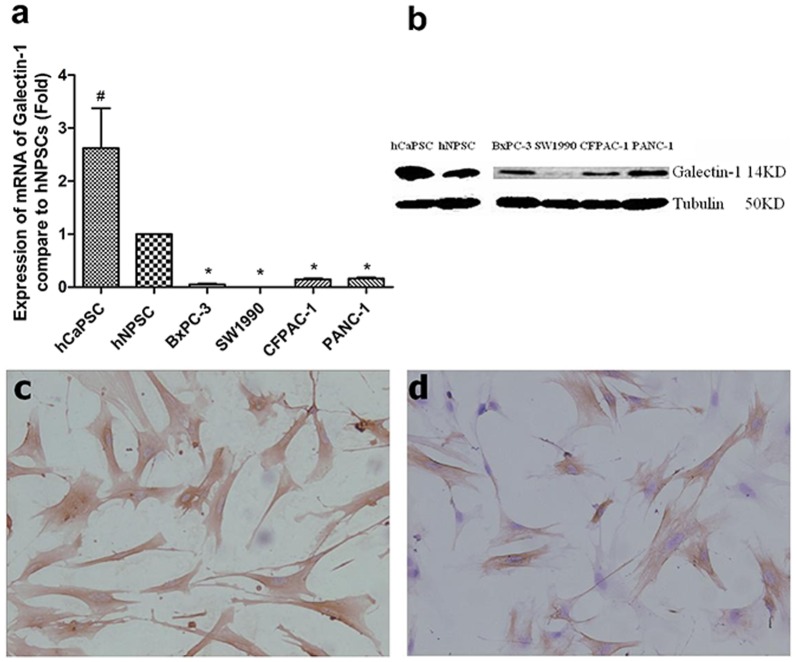
Expression of Galectin-1 in primary isolated PSCs and pancreatic cancer cell lines (BxPC-3, SW1990, CFPAC-1 and PANC-1). (*a*) Quantitative analyses of the differentiated phenotype of human PSCs and pancreatic cancer cell lines. Higher Galectin-1 mRNA expression was observed in the hCaPSC, and lower Galectin-1 mRNA expression was observed in pancreatic cancer cell lines. *P<0.01 vs. hNPSC, ^#^P<0.05 vs. hNPSC. hNPSC, PSC isolated from normal pancreas tissue; hCaPSC, PSC isolated from pancreatic cancer tissue. (*b*) Typical Western blot indicating stronger band of Galectin-1 in hCaPSC and hNPSC than that in pancreatic cancer cell lines. (*c*) Strong immunohistochemisty staining of Galectin-1 in hCaPSC. (*d*) Moderate immunohistochemisty staining of Galectin-1 in hNPSC. Original magnification: ×200.

To determine the effect of PSCs derived Galectin-1 on the progression of PDAC, we isolated human cancer PSCs (hCaPSC) and normal PSCs (hNPSC) from the resected pancreatic cancer tissue and the normal pancreas tissue of the patient undergoing operation for bile duct cancer respectively. After cultured for 10 days, total RNA was extracted by Trizol and the mRNA of Galectin-1 were detected by qRT-PCR. The results demonstrated that the mRNA of Galectin-1 in PSCs were significantly higher than that in pancreatic cancer cell lines (BxPC-3, SW1990,CFPAC-1 and PANC-1), and the mRNA of Galectin-1 in hCaPSC was also significantly higher than the hNPSC (Figure 3 *a*), which were coincident with the results of immunohistochemistry (Figure 1). The Galectin-1 band corresponding to hCaPSC was also markedly more prominent than that in hNPSC (Figure 3 b). All the results indicated that the Galectin-1 was mainly expressed in cancer-associated PSCs.

### PSCs Derived Galectin-1 Promote the Proliferative Activity (S-phase fraction) of CFPAC-1 cells

**Figure 4:**
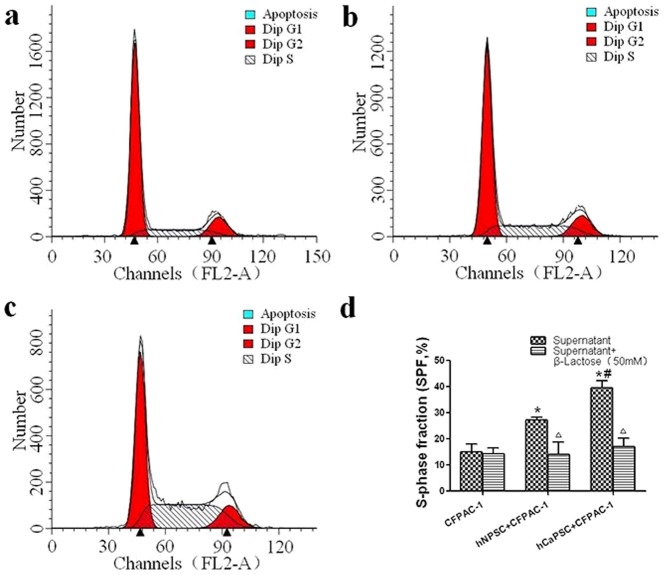
PSCs derived Galectin-1 promotes the proliferative activity (S-phase fraction) of CFPAC-1 cells. (a–c) Quantitative graphical representation of Apoptosis, G1, G2 and S cell population. Points, average of three independent experiments. a, CFPAC-1; b, CFPAC-1 + hNPSC; c, CFPAC-1 + hCaPSC. (d) A bar-graphical representation S-Phase Fraction cells in each group. *p<0.01 *vs*. CFPAC-1, #*p*<0.05 *vs*. hNPSC+CFPAC-1, ^△^ *p*>0.05 *vs*. hNPSC +β-Lactose.

To determine whether the PSCs derived Galectin-1 induced the proliferative activity of pancreatic cancer cells, we analyzed the S-phase fraction (SPF) of CFPAC-1 by flow cytometry, and the percentages of cells in Apoptosis, G1, S, and G2 were quantified. The mean SPF value in the group of CFPAC-1 co-cultured with hNPSC was 0.27±0.59% and it was significantly higher (p = 0.003) than that of control subjects (CFPAC-1 only, 0.15±1.80%). However, mean SPF value in the group of CFPAC-1 co-cultured with hCaPSC was 0.40±1.53% and it was significantly higher (p<0.001) than that of control subjects, and also significantly higher (p = 0.002) than that group of CFPAC-1 co-cultured with hNPSC (Figure 4).

To further verify the finding that endogenous Galectin-1 promotes the proliferative activity of the CFPAC-1, β-Lactose (50 mM) were added as the antagonist to Galectin-1 in three groups. As the results, the β-Lactose reduced the SPF to 0.14±1.31%, 0.14±2.75%, 0.17±1.89% in the groups of CFPAC-1, CFPAC-1 + hNPSC and CFPAC-1 + hCaPSC (p>0.05), respectively (Figure 4). This finding indicated that β-lactose, as a competitive inhibitor of Galectin-1, completely blocked the ability of endogenous Galectin-1 that promote the proliferative activity of the CFPAC-1, confirming the effect that increased proliferative activity of CFPAC-1 cells was specifically induced by PSC derived Galectin-1.

### Endogenous Galectin-1 of PSC Upregulated by TGF-β1 and Induced MMP2 and MMP9 Expression, and Promotes the Invasion of CFPAC-1

**Figure 5:**
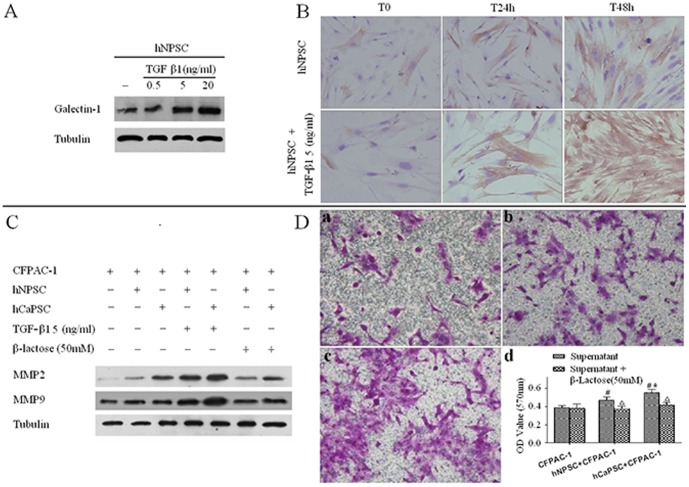
Endogenous Galectin-1 of PSC upregulated by TGF-β1 and induced MMP2 and MMP9 expression, and promotes the invasion of CFPAC-1. (A) TGF-β1 regulates Galectin-1 expression in primary cultured hNPSCs. hNPSCs were treated for 24 hours with different concentrations (0.5, 5 and 20 ng/ml) of TGF-β1, which was found induced in a dose-dependent increase of Galectin-1 expression in hNPSCs. (B) TGF-β1 upregulates Galectin-1 expression in the cytoplasm of hNPSCs. Subcellular distribution of Galectin-1 upon TGF-β1 treatment was evaluated by immunohistochemical staining. hNPSCs were treated for 24 and 48 hours with 5 ng/ml TGF-β1 or left untreated. Original magnification: ×200. (C) PSCs derived Galectin-1 increased the expression of MMP-2 and MMP-9 in PCCs. Expression of MMP-2and MMP-9 in PCCs was analyzed by western blot. hCaPSC increased more expression of MMP-2 and MMP-9 in CFPAC-1 than that of hNPSCs, and both of hCaPSC and hNPSCs significantly elevated the expression of both MMP-2and MMP-9 when add 5 ng/ml of TGF-β1 into the supernatant, but β-lactose (50 mM) competitively inhibiting Galectin-1 reduced them. (D)Transwell invasion assay of the pancreatic cancer cell line CFPAC-1. D-a. Invasion of CFPAC-1 in transwell chambers by serum free medium. D-b. Invasion of CFPAC-1 stimulated by hNPSC in transwell chambers. D-c. Invasion of CFPAC-1 stimulated by hCaPSC in transwell chambers. D-d. OD value of each group of invaded CFPAC-1 cells with or without β-Lactose (50 mM). #p<0.05 vs. CFPAC-1, *p<0.05 vs. hNPSC+CFPAC-1, △p<0.05 vs. hNPSC+β-Lactose.

Two critical mediators of TGF-β1 and Galectin-1 have a potential cross talk during the cancer cells progression and tumor-immune escape [28], but the extensional role of which on the interrelation between PSCs and PCCs in PDAC has not yet well elucidated. Here, we first investigated the Galectin-1 expression on the primary culture of hNPSCs by the regulation of ectogenesis TGF-β1. Primary culture of hNPSCs was treated for 24 hours with increasing concentrations of TGF-β1. The results indicated that TGF-β1 induced in a dose-dependent increase in Galectin-1 expression in hNPSCs (Figure 5 A). To illustrate the morphologically cellular distribution of Galectin-1 with a time-sequence manner, hNPSCs were treated with TGF-β1 for 24 and 48 hours, and analyzed by immunohistochemical staining. As shown in Figure 5 B in the absence of TGF-β1 (T0) hNPSCs displayed a weak Galectin-1 staining mostly in the cytoplasm. Compare to the normal control, treatment of hNPSCs with TGF-β1 (5 ng/ml) obviously increased Galectin-1 staining in a time-dependent fashion (Figure 5 B). In addition, the normal control of hNPSCs also slightly increased Galectin-1 staining in a time-dependent which may indicated the internal activate mechanism. As PDAC cells can secrete cytokines TGF-β1 [29], our results suggested that PCCs may upregulated Galectin-1 expression in hNPSCs by paracrine secretion of TGF-β1.

Matrix metalloproteinases (MMPs) are a family of zinc-dependent enzymes that are able to degrade ECM proteins and promote tumor cell invasion and metastasis [20]. Therefore, we further examined whether endogenous Galectin-1 derived from PSCs affects MMP-2 and MMP-9 expression. Co-cultured of PSCs and CFPAC-1 by transwell were used, and the results showed that hCaPSC increased more expression of MMP-2 and MMP-9 in CFPAC-1 than that of hNPSCs, and both of hCaPSC and hNPSCs significantly elevated the expression of both MMP-2and MMP-9 when add 5 ng/ml of TGF-β1 into the supernatant, but β-lactose (50 mM) competitively inhibiting Galectin-1 reduced them (Figure 5 C). In addition, with the presence of hNPSC or hCaPSC in the lower transwell chambers, the invasion ability of CFPAC-1 was significantly enhanced (p = 0.019 vs. control, p = 0.02 vs. control, respectively), and the invasion ability of CFPAC-1 co-cultured with hCaPSC was significantly increased than that co-cultured with hNPSC (p = 0.039), and the effects could be significantly weakened by β-Lactose (50 mM) added in the lower chamber (p>0.05 vs. control) (Figure 5 D). This showed that PSCs derived Galectin-1 promoted PCCs invasion partly by augmenting MMP-2 and MMP-9 expression.

### Effect of PSCs Derived Galectin-1 on Tumor Establishment and Growth

**Figure 6:**
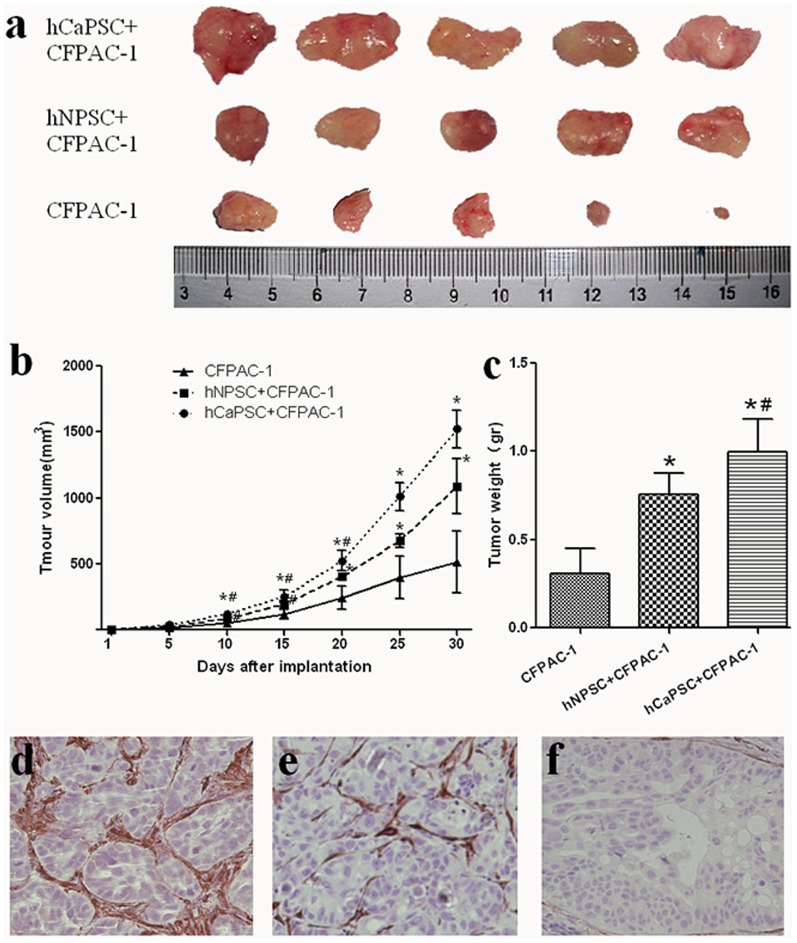
Effect of PSCs derived Galectin-1 on tumor establishment and growth. (a) CFPAC-1 with or without hCaPSC, hNPSC was implanted subcutaneously on both flanks of nude mice (n = 5), and the mice were sacrificed and xenografts were take out on day 30 after cell implantation. Tumor volume (b) and weight (c) is expressed as the mean±SE. *p<0.01 vs. CFPAC-1, ^#^p<0.05 vs. hNPSC+CFPAC-1. Immunohistochemisty staining of Galectin-1 in the xenografts were performed in the group of hCaPSC+CFPAC-1(d), hNPSC+CFPAC-1(e) and CFPAC-1(f). Original magnification: ×200.

In order to validate the effect of PSCs derived Galectin-1 on PCCs in vivo, CFPAC-1 cells were implanted subcutaneously with hNPSC or hCaPSC. The volume and weight of tumor reached was prone to increased significantly compare to the control group (CFPAC-1 only) from the day 10 (Figure 6 *a-c*). 30 days after cell implantation, tumors of group CFPAC-1 + hCaPSC reached a volume of 1521.4±62.5 mm^3^ and weight of 0.998±0.083 g, and tumors of group CFPAC-1 + hNPSC reached a volume of 1089.0±94.0 mm^3^ and weight of 0.758±0.052 g, while tumors of control group only reached a volume of 516.0±104.5 mm^3^ and weight of 0.310±0.064 g, respectively. All the results showed that the tumors of group CFPAC-1+ hCaPSC grew faster and bigger than that of group CFPAC-1+hNPSC (p<0.001) and control group (p<0.01), and the tumors of group CFPAC-1+ hNPSC also grew faster and bigger than that of control group (p<0.01). When all the tumor was analyzed by immunohistochemical staining, we found that tumors of group CFPAC-1 + hCaPSC contained higher levels of Galectin-1 in the PSCs around the CFPAC-1 pancreatic tumor cells than that of group CFPAC-1 + hNPSC (Figure 6 *d-f*), and almost no Galectin-1 staining was observed in the tumors of control group. All of these indicated PSCs derived Galectin-1 promote the tumor establishment and growth.

## Discussion

This study focused on evaluating the Galectin-1 function in PDAC development. We showed that Galectin-1 protein was gradually increased from normal pancreas, chronic pancreatitis to PDAC, and in PDAC Galectin-1 protein expression was also increased from well, moderately to poorly differentiated PDAC. Even in the metastatic lymph nodes were found Galectin-1 positive straining. Furthermore, PSCs derived Galectin-1 promotes the proliferative activity, MMP2 and MMP9 expression and invasion of pancreatic cancer cells in vitro, and tumor establishment and growth in vivo. Taken together, the results suggest that Galectin-1 may serve as a potential biomarker to predict the risk for PDAC development.

Galectin-1 is differentially expressed by various normal and pathological tissues and appears to be functionally polyvalent, with a wide range of biological activity, including modulation of cells apoptosis, migration, adhesion, malignant transformation, tumor angiogenesis and tumor immunosuppression[12], [30], [31], [32], [33]. Regarding PDAC, Galectin-1 has been proved to be a PDAC related protein[8], [17], [34]. Galectin-1 was expressed in the activated PSCs which were the source of fibroblasts around pancreatic cancer cells, and have a role in chemokine production and proliferation through its beta-galactoside binding activity in activated PSCs[9], [17], [35].

PSCs are the main source of extracellular matrix synthesis that leading to pancreatic fibrosis and are activated by growth factors, inflammatory cytokines, alcohol, its metabolite acetaldehyde and oxidative stress[36], [37], [38]. Our immunohistochemical results indicated that increased Galectin-1 staining in chronic pancreatitis was found significant correlation with alcohol drinking, which have been proved to able to activate the PSCs and result in Galectin-1 expression[39], [40]. More important, Galectin-1 was strong expressed in pancreatic cancer, of which Galectin-1 expression was closely related to tumor size, perineural invasion, tumor stage, differentiation and poor prognosis. In addition, we also found that the metastatic peripancreatic lymph nodes existed Galectin-1 positive straining, which have not well observed in other researches and might be explained as follows: first, the high Galectin-1 expressed in stromal cells, especially activated PSCs, induced epithelial-mesenchymal transition (EMT) in the pancreatic cancers cells[41]; second, the high level of Galectin-1 in PSCs promoted the cancer cells to acquire a metastatic phenotype[12], [42], accordingly the cancer cells itself increased the Galectin-1 expression and acquired an advanced progression. So, it will be well accepted that Galectin-1 expression or overexpression in tumors and/or the tissue surrounding them must be considered as a sign of the malignant tumor progression[12].

**Figure 7:**
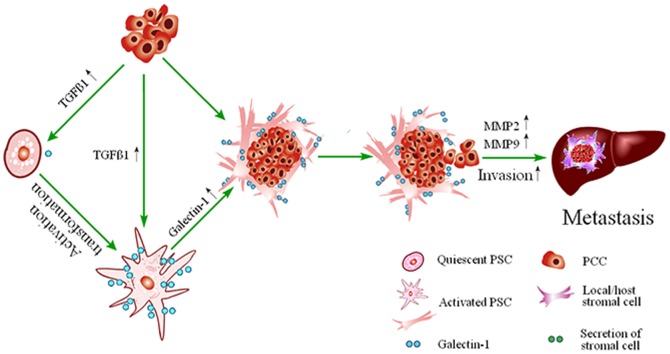
Mechanism of hCaPSCs derived Galectin-1 increased the invasion and metastasis of PDCA. By secreting the cytokine of TGF-β1, PDAC cells stimulate the activation of quiescent PSCs and promote PSCs to express high level of Galectin-1. Galectin-1 has also been involved in PSCs activation, proliferation and chemokine production, which in turn stimulate the malignant potency of pancreatic cancer cells by increased both the expression of MMP2 and MMP9, and then establish a vicious cycle of mutually reinforcing mechanisms to sustain the activity of the stromal reaction and promotes the invasion and metastasis of PDCA.

In order to further confirm the role of PSCs derived Galectin-1 on pancreatic cancer progression, we isolated PSCs from pancreatic cancer tissues (hCaPSCs) and normal pancreatic tissue (hNPSC), respectively. We found that Galectin-1 mRNA and protein levels were significantly higher in isolated cultured PSCs compared to pancreatic cancer cell lines (BxPC-3, CFPAC-1, SWl990 and PANC-1), which is consistent with the immunohistochemisty results that Galectin-1 mostly expressed in cancer-associated stromal cells. Furthermore, PSCs derived Galectin-1 was upregulated by TGF-β1, and in turn induced the invasion of PCCs by elevating the expression of both MMP-2and MMP-9, which may further promote the invasion and metastasis of PCCs. In addition, pancreatic cancer cells implanted subcutaneously alone produced small tumors, which progressed slowly; whereas addition of PSCs to the implanted cancer cells resulted in larger tumors, which progressed much faster. The size of tumors produced and speed of their development did not depend solely on the presence of the PSCs, but also the expression levels of PSCs derived Galectin-1, because cancer cells that were implanted together with pancreatic cancer associated PSCs (hCaPSCs) that exhibited high levels of Galectin-1 resulted in larger volume and weight of tumors, than those that were implanted with PSCs from normal pancreas. In brief, by secreting the cytokine of TGF-β1 [29], PDAC cells stimulate PSCs to express high level of Galectin-1. Galectin-1 has also been involved in PSCs activation, proliferation and chemokine production[9], [35], which in turn stimulate the malignant potency of pancreatic cancer cells and establish a vicious cycle of mutually reinforcing mechanisms to sustain the activity of the stromal reaction[43], [44] and promotes the malignant behavior of PDAC (illustrated as Figure 7).

Up to now, several studies have evaluated the correlation between Galectin-1 expression and PDAC[8], [17], [18], [45], [46], [47]. Gene expression profiling of pancreatic ductal carcinomas indicated that Galectin-1 is one of most highly expressed genes in PDAC[34], [45]. Berberat et al[17] reported that the expression pattern of Galectin-1 in pancreatic cancer tissues indicated that Galectin-1 played a key role in the desmoplastic reaction that occurred around pancreatic cancer cells, and expressed significantly higher in G3 tumors than that in G1+2 tumors, but had no significant relationship with the presence of metastasis and postoperative survival. While, Chung et al[48] perform a proteomic analysis between normal pancreas and PDAC and found Galectin-1 expression highly correlated to histology, T stage, N stage and AJCC stage. In agreement with findings of the other study [47], we found that staining intensity of Galectin-1 in stromal cells were significant associated with short patient survival, tumor stage and lymph node metastasis. In addition, the in vivo and vitro experiments also verified the PSCs derived Galectin-1 might increase the reproductive activity and invasion ability of pancreatic cancer cells[46], and facilitate the growth of the subcutaneous xenografts. As only 10 and 33 cases of pancreatic cancer specimens were reported by Chung et al[48] and Berberat et al[17], the discrepancies among our study and the existed reports might ascribe to the different number of specimen and the evaluation approach. However, existent evidence indicated that PSCs derived Galectin-1 might play an important role on the progression of PDAC, and there is a good prospect that it is associated with several aspects as follows: First, Galectin-1 supports metastasis formation, because it facilitates interactions between tumor cells and endothelium cells[49], [50], mediates cell-cell or cell extracellular matrix adhesion[51], promotes cancer cell migration, growth, and metastasis[52]. Second, it protects the tumor against immunity, because it can induce apoptosis in tumor-infiltrating cytotoxic leukocytes[53], [54]. Third, it palys a critical role on tumor angiogenesis, which is an important pillar in tumor progression[32], [55].

In conclusion, our findings supported that expression of Galectin-1 inPSCs was related with tumor invasiveness and progression as well as with short patient survival in pancreatic cancer. PSCs derived Galectin-1 seem to be the key elements in the cross-talk between the parenchymal cells and the desmoplastic stroma,which may enhance metastatic potential of tumor cells and result in poor prognosis. Galectin-1 may be a promising molecular target for the development of new and original therapeutic tools. However,the limitations of this study is that the relationship between PSCs derived Galectin-1 and the PDAC is only a descriptive work, and the specific mechanism of PSCs derived Galectin-1 promote the progression of PDAC needs further clarification by the method of gene transfection to knock down and up-regulate the Galectin-1 in PSCs.
